# Incidence of Acute Kidney Injury Post Transcatheter Aortic Valve Implantation (TAVI): A Single-Center Experience

**DOI:** 10.7759/cureus.65187

**Published:** 2024-07-23

**Authors:** Khalid Makki, Fatemah I Ammar, Jose Andres Fernandez, Muhnnad A AlGhamdi, Abdulkareem M Alturkistani, Rahaf A Hubayni, Elaf I Khahwry

**Affiliations:** 1 Department of Anesthesiology, King Faisal Cardiac Center, Ministry of National Guard Health Affairs, Jeddah, SAU; 2 College of Medicine, King Saud Bin Abdulaziz University for Health Sciences, Jeddah, SAU; 3 Department of Research, King Abdullah International Medical Research Center, Jeddah, SAU; 4 College of Medicine, King Abdulaziz University, Jeddah, SAU

**Keywords:** contrast media, estimated glomerular filtration rate, chronic kidney disease, transcatheter aortic valve implantation, acute kidney injury

## Abstract

Introduction: Transcatheter aortic valve implantation (TAVI) has dramatically changed the approach to treating aortic stenosis, particularly for patients unsuitable for surgical aortic valve replacement. Nevertheless, the possibility of quick deterioration of kidney function, known as acute kidney injury (AKI), post operation is considered one of the complications.

Objectives: The study aimed to determine the incidence of AKI in adults post TAVI.

Methods: This retrospective cohort study focuses on patients who underwent the TAVI procedure at the King Faisal Cardiac Center at the Ministry of National Guard Health Affairs (MNGHA) in Jeddah, Saudi Arabia, from May 2016 to December 2022. Acute kidney injury post TAVI was defined based on RIFLE (Risk, Injury, Failure, Loss of kidney function, and End-stage kidney disease) criteria. Chi-square tests and independent sample t-tests were used to compare clinical and demographic characteristics between patients who developed AKI with those who did not, using an alpha of 5%.

Results: The study involved 103 adult patients. Among them, 11 (10.7%) developed AKI post TAVI within seven days of the procedure, while 92 (89.3%) did not. Findings also revealed that patients with hyperlipidemia and a previous history of kidney diseases faced a higher risk of AKI post TAVI. Despite its valuable insights, the study has limitations due to its retrospective nature and small sample size.

Conclusions: The study emphasizes the importance of identifying patients with hyperlipidemia and pre-existing kidney conditions and closely monitoring renal function. While some preventive methods did not significantly impact AKI occurrences, further research is needed to refine preventive strategies.

## Introduction

Acute kidney injury (AKI) is a condition characterized by the rapid deterioration of kidney function, leading to electrolyte and volume imbalances [1]. Alongside AKI, another common condition in the elderly population is aortic valve stenosis. Aortic stenosis, characterized by the narrowing of the aortic valve, typically occurs as a result of the aging process. However, it can also result from congenital defects or, rarely, rheumatic fever [2]. Recently, transcatheter aortic valve implantation (TAVI) has emerged as a viable alternative for patients with severe aortic stenosis who are considered ineligible for conventional surgical aortic valve replacement [3]. The American Heart Association defines TAVI as a minimally invasive percutaneous procedure that helps to improve a damaged aortic valve by replacing it through an endovascular technique [4]. However, it is crucial to note that AKI following TAVI is a recognized complication [5]. Post-contrast AKI (PC-AKI) is a well-established risk following procedures involving contrast, such as TAVI. The variation in the reported incidences of AKI post TAVI is influenced by factors such as the absence of a standardized definition, different routes of access, investigation tools, and comorbidities. The reported incidence of AKI post TAVI varies widely due to different definitions and patient factors [6, 7]. These causes influence the variation in the reported incidence of AKI, which ranges between 8.3% and 58% [7]. Another study by Alassar et al. [8] found an AKI incidence rate after TAVI of 12.3% that resolved before patients’ discharge from the hospital. Also, AKI occurred in 13.8% of TAVI patients in Haase-Fielitz et al. [9]. Given the common occurrence and severe consequences of AKI complicating TAVI, a standardized PC-AKI definition is crucial [10]. The RIFLE (Risk, Injury, Failure, Loss of kidney function, and End-stage kidney disease) criteria use estimated glomerular filtration rate (eGFR) changes, adjusted for age and gender, to estimate AKI incidence and identify early-stage patients [11]. The Kidney Disease Improving Global Outcomes (KDIGO) criteria define AKI as a 0.3 mg/dL or 1.5-1.9 times increase in serum creatinine (sCr) from baseline, providing reliable incidence and outcome estimates [12-14].

Predictive factors for AKI following TAVI include age, diabetes mellitus, heart failure, atherosclerotic burden, previous stroke, and chronic kidney disease (CKD) [6]. Acute kidney injury post TAVI can lead to serious complications such as permanent renal dysfunction requiring renal replacement therapy, heart attack, life-threatening hemorrhage, and mortality [15,16]. Despite the risks, certain studies have found TAVI to be safe when specific techniques are employed [17, 18]. Procedure-related factors that increase the risk of PC-AKI include the type, volume, and route of contrast administration, as well as the frequency of injections. Peri-procedural intravenous hydration remains a key preventive strategy [19]. Effective prevention involves identifying at-risk patients and administering rigorous hydration with non-ionic contrast [20]. Healthcare practitioners should evaluate each patient's risk to determine the appropriate hydration approach [20, 21]. This research aimed to estimate the occurrence of AKI in adult patients who underwent the TAVI procedure. 

## Materials and methods

Study population

**Figure 1:**
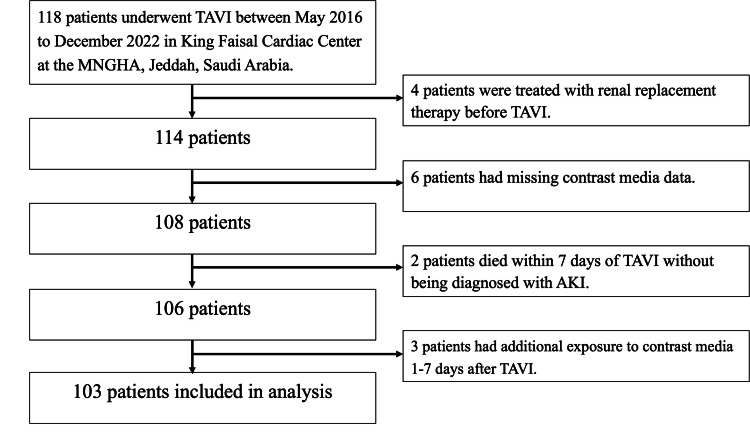
Patient selection flowchart TAVI: transcatheter aortic valve implantation; AKI: acute kidney injury; MNGHA: Ministry of National Guard Health Affairs

This study utilized a retrospective cohort design to estimate the incidence of AKI in adult patients post TAVI procedure in the King Faisal Cardiac Center in the Ministry of National Guard Health Affairs (MNGHA) at King Abdulaziz Medical City (KAMC), Jeddah, Saudi Arabia. The study included 103 adult patients. The patient selection flowchart is shown in Figure 1.

Inclusion and exclusion criteria

This study included adult patients from the King Faisal Cardiac Center at MNGHA who were 14 years of age or older and had undergone the TAVI procedure during the designated study period. At MNGHA, patients who are 14 years old or older are considered adults. Patients were excluded if they had a history of exposure to contrast medium within the first seven days post TAVI if any pre- or postoperative measurement of sCr or contrast medium volume was missing, or if they had died within the first seven days. Additionally, patients who had a kidney transplant were on maintenance hemodialysis or peritoneal dialysis or had preexisting moderate to severe kidney damage stage 3b CKD and further, defined as a decreased eGFR of less than 30 mL/min/1.73 m^2^ were also excluded [22].

Data collection

Data were collected from the medical records department through the Best Care system for all patients in the cardiology department who underwent the TAVI procedure from May 2016 to December 2022. Demographic data and sCr levels of each participant were used to calculate eGFR using the Chronic Kidney Disease Epidemiology Collaboration (CKD-EPI) creatinine equation (2021) [23]. Acute kidney injury post TAVI was defined based on the RIFLE criteria: the risk stage as sCr increased to 1.5-fold or GFR decreased by >25% from baseline; the injury stage as sCr increased to 2.0-fold or GFR decreased by >50% from baseline; and the failure stage as sCr increased to 3.0-fold or GFR decreased by >75% from baseline, within two to seven days post TAVI [11, 24]. Laboratory data were measured one day before the procedure (pre-TAVI), on the procedure day (procedure date), and seven days after the procedure (post-TAVI). The RIFLE urine output criteria were excluded from defining AKI due to the use of diuretic medication, which may affect the results [11]. Additionally, according to hospital protocol, patients with heart failure received prophylactic hydration with isotonic saline 12 hours before TAVI, while the remaining patients received individualized prophylactic hydration. The Mehran score was used to further classify the risk of contrast-induced nephropathy after TAVI [25].

Statistical analysis

Categorical variables were reported as absolute numbers and percentages and compared using the chi-square test. Continuous data with a normal distribution were reported as the mean and standard deviation (SD) and compared using an independent sample t-test. A p-value lower than 0.05 was considered statistically significant; data were managed and analyzed in IBM SPSS Statistics software for Windows, version 29.0 (IBM Corporation, Armonk, NY).

## Results

**Figure 2:**
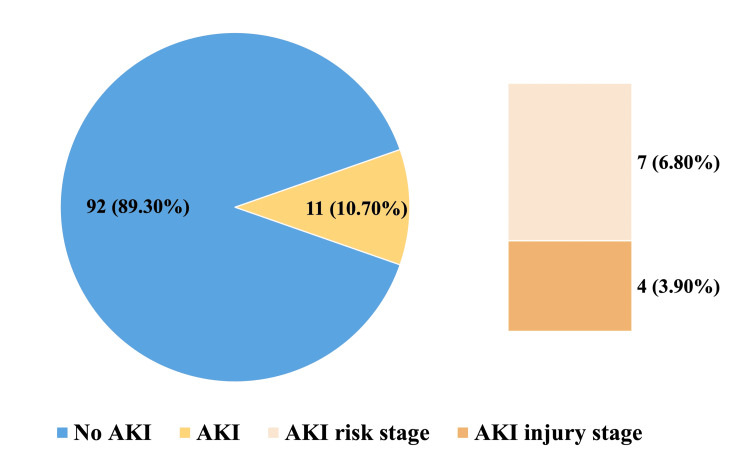
Distribution of patients with and without AKI according to the RIFLE criteria AKI: acute kidney injury; RIFLE: Risk, Injury, Failure, Loss of kidney function, and End-stage kidney disease

The study included 103 adult patients, with 92 (89.3%) patients in the AKI (-) group and 11 (10.7%) in the AKI (+) group. Out of the 11 patients with AKI (+), seven (6.8%) patients were in the risk stage, and four (3.9%) patients were in the injury stage. Figure 2 displays the distribution of patients with and without AKI according to the RIFLE criteria. 

Equations

The calculation of the incidence proportion of patients who are diagnosed with AKI following TAVI procedures is shown below.

$\large Incidence\, proportion=\tfrac{Number\, of\, new\, cases\, of\, the\, disease}{Population\, at\, risk\, over\, a\, specified\, time\, period}\times 100$

$\large Incidence\, proportion=\tfrac{AKI\, cases\, (11)}{Total\, population\, at\, risk\, (103)}\times 100=10.7\%$%

Baseline characteristics 

The study included 103 patients, of whom 52 were male and 51 were female. Among the male patients, four (7.7%) out of 52 male patients and seven (13.7%) out of 51 female patients developed AKI. The mean age of patients who developed AKI was 82.18 ± 7.82 years, while the mean age of patients who did not develop AKI was 78.66 ± 9.24 years. The average body mass index (BMI) was 32.20 ±4.99 kg/m^2^ for patients who developed AKI and 30.79 ±7.65 kg/m^2^ for patients who did not develop AKI. In terms of comorbidities, type 2 diabetes mellitus was present in 54.5% of the patients who developed AKI post TAVI; hypertension was present in all patients who developed AKI post TAVI, and dyslipidemia was seen in 81.8%. Six (54.5%) patients had heart failure, and CKD was described in nine (81.8%) patients who developed AKI. The baseline characteristics (Table 1) showed no statistically significant differences between the AKI (-) and AKI (+) groups in terms of gender, age, BMI, diabetes mellitus, hypertension, anemia, smoking, heart failure, malignancy, infection, bleeding, multi-organ disease, and nephrotoxic drugs. However, patients with hyperlipidemia and a history of kidney disease had a significantly higher risk of developing AKI following TAVI. Therefore, those with hyperlipidemia or prior kidney disease are more at risk of developing AKI following TAVI.

**Table 1:** Baseline characteristics of the study group AKI: acute kidney injury; NSAIDs: non-steroidal anti-inflammatory drugs

Clinical data		AKI (-); n (%)	AKI (+); n (%)	p-value
1. Gender	Male	48 (52.2%)	4 (36.4%)	0.322
	Female	44 (47.8%)	7 (63.6%)	
2. Age (years) mean ± SD		78.66 ± 9.24	82.18 ± 7.82	0.229
3. Body mass index (kg/m^2^) mean ± SD		30.79 ±7.65	32.20 ± 4.99	0.421
4. Diabetes mellitus	Yes	59 (64.1%)	6 (54.5%)	0.529
	No	33 (35.9%)	5 (45.5%)	
5. Hypertension	Yes	75 (81.5%)	11 (100.0%)	0.204
	No	17 (18.5%)	0 (0.0%)	
6. Hyperlipidemia	Yes	66 (71.7%)	9 (81.8%)	0.025
	No	23 (25.0%)	0 (0.00%)	
	Not documented	3 (3.3%)	2 (18.2%)	
7. Anemia	Yes	41 (44.6 %)	6 (54.5%)	0.543
	No	51 (55.4%)	5 (45.5%)	
8. Smoking	Yes	7 (7.6%)	0 (0.0%)	0.802
	No	51 (55.4%)	7 (63.6%)	
	Not documented	27 (29.3%)	3 (27.3%)	
	Ex-smoker	7 (7.6 %)	1 (9.1%)	
9. Heart failure	Yes	26 (28.3%)	6 (54.5%)	0.092
	No	66 (71.7%)	5 (45.5%)	
10. Kidney disease	None	73 (79.3%)	2 (18.2%)	<0.001
	History of AKI	2 (2.2%)	0 (0.0%)	
	CKD	15 (16.3%)	9 (81.8%)	
	Others	2 (2.2%)	0 (0.0%)	
11. Malignancy	Yes	14 (15.2%)	3 (27.3%)	0.385
	No	77 (84.8%)	8 (72.7%)	
12. Infection	Yes	8 (8.7%)	2 (18.2%)	0.289
	No	84 (91.3%)	9 (81.8%)	
13. Bleeding	Yes	2 (2.2%)	1 (9.1%)	0.290
	No	89 (97.8%)	10 (90.9%)	
14. Multi-organ disease	Yes	8 (8.7%)	2 (18.2%)	0.289
	No	84 (91.3%)	9 (81.8%)	
15. Nephrotoxic drugs	None	35 (38.0%)	4 (36.4%)	0.837
	NSAIDs	40 (43.5%)	4 (36.4%)	
	Antimicrobial agents	4 (4.3%)	0 (0.0%)	
	Chemotherapeutic agents	4 (4.3%)	1 (9.1%)	
	Antimicrobial and chemotherapeutic agents	1 (1.1%)	0 (0.0%)	
	Others drugs	8 (8.7%)	2 (18.2%)	

Laboratory data

Laboratory data analysis (Table 2) indicated no significant differences in pre-TAVI laboratory tests. However, significant changes were observed in sCr, eGFR, and blood urea nitrogen (BUN) levels on the procedural date and post TAVI. On the TAVI day, sCr (p=0.001), eGFR (p<0.001), and BUN (p=0.025) were significantly affected, and post TAVI, the significance persisted for sCr (p=0.006), eGFR (p<0.001), and BUN (p=0.002). These results suggest that contrast media significantly impacted kidney function, particularly in patients with a history of kidney diseases. 

**Table 2:** Laboratory data AKI: acute kidney injury; TAVI: transcatheter aortic valve implantation; pre-TAVI: one day before the procedure; procedure date: on the day of the TAVI procedure; post-TAVI: seven days after the procedure

Laboratory test		AKI (-)	AKI (+)	p-value
Hemoglobin (gm/dL), mean (SD)	Pre-TAVI	12.25 (1.74)	11.93 (1.16)	0.569
	Procedure date	12.97 (10.64)	11.60 (1.42)	0.673
	Post-TAVI	11.04 (1.74)	10.56 (1.51)	0.383
White blood count (*10^9/L), mean (SD)	Pre-TAVI	6.91 (1.81)	7.74 (1.54)	0.154
	Procedure date	8.18 (3.23)	10.78 (5.52)	0.023
	Post-TAVI	9.10 (2.73)	12.26 (4.49)	0.001
Platelet (*10^9/L), mean (SD)	Pre-TAVI	241.46 (67.85)	255.27 (77.12)	0.531
	Procedure date	235.45 (63.48)	232.55 (81.15)	0.890
	Post-TAVI	206.01 (81.66)	186.55 (87.00)	0.460
Sodium (mmol/L), mean (SD)	Pre-TAVI	136.17 (3.58)	136.91 (2.30)	0.508
	Procedure date	136.16 (3.52)	136.81 (3.62)	0.567
	Post-TAVI	133.86 (12.09)	135.61 (3.78)	0.636
Potassium (mmol/L), mean (SD)	Pre-TAVI	4.38 (0.47)	4.64 (0.61)	0.106
	Procedure date	4.48 (1.96)	4.49 (0.62)	0.992
	Post-TAVI	4.29 (0.48)	4.49 (1.14)	0.570
Albumin (g/L), mean (SD)	Pre-TAVI	37.14 (4.26)	36.50 (4.25)	0.661
	Procedure date	35.42 (3.99)	35.00 (4.57)	0.785
	Post-TAVI	34.32 (3.48)	30.89 (5.01)	0.078
Serum creatinine (umol/L), mean (SD)	Pre-TAVI	88.52 (28.78)	116.82 (43.86)	0.061
	Procedure date	84.83 (26.86)	115.09 (37.35)	0.001
	Post-TAVI	83.91 (29.10)	164.36 (77.28)	0.006
Blood urea nitrogen (mmol/L), mean (SD)	Pre-TAVI	9.06 (14.73)	10.04 (3.55)	0.828
	Procedure date	7.29 (3.75)	9.95 (2.82)	0.025
	Post-TAVI	8.06 (4.92)	13.01 (4.94)	0.002
Estimated glomerular filtration rate (eGFR), mean (SD)	Pre-TAVI	69.87 (18.83)	59.09 (20.30)	0.078
	Procedure date	71.18 (18.34)	48.64 (17.81)	< 0.001
	Post-TAVI	73.72 (19.04)	34.64 (14.81)	< 0.001
Prothrombin time (PT) (s), mean (SD)	Pre-TAVI	12.77 (1.52)	13.82 (2.56)	0.212
	Procedure date	13.27 (1.86)	14.09 (1.51)	0.164
	Post-TAVI	13.64 (3.93)	16.91 (12.68)	0.415
Partial thromboplastin time (PTT) (s), mean (SD)	Pre-TAVI	31.99 (5.51)	34.82 (5.76)	0.112
	Procedure date	37.37 (23.05)	51.18 (35.72)	0.081
	Post-TAVI	32.87 (7.86)	34.00 (7.94)	0.654
International normalised ratio (INR), mean (SD)	Pre-TAVI	1.13 (0.16)	1.23 (0.16)	0.013
	Procedure date	1.09 (0.13)	1.21 (0.24)	0.072
	Post-TAVI	1.17 (0.33)	1.49 (1.17)	0.389

Prophylaxis methods and contrast media data

All patients who underwent TAVI received prophylaxis to prevent kidney damage (Table 3). This involved hydration administered orally, intravenously, or both. The findings indicate that the type of hydration did not significantly affect outcomes for either the AKI (-) or AKI (+) groups (p = 0.795). Additionally, hydration administered before and after contrast media exposure also showed no significant impact on outcomes (p = 0.121). Furthermore, all patients received iodinated iso-osmolar and isotonic contrast media administered intraarterially in the contrast media data. The volume of contrast media administered did not yield a significant outcome (p = 0.527). Also, analyzing the Mehran score for patients categorized as low-risk, moderate-risk, high-risk, and very high-risk showed no significant difference (p = 0.120). Therefore, no significant association was found between the volume of contrast media administered, the Mehran score, and the occurrence of AKI.

**Table 3:** Prophylaxis methods and contrast media data AKI: acute kidney injury; TAVI: transcatheter aortic valve implantation

		AKI (-); n (%)	AKI (+); n (%)	p-value
Hydration type	Oral	8 (100.0 %)	0 (0.0%)	0.795
	Intravenous (IV)	33 (86.8 %)	5 (13.2%)	
	Both	51 (89.5 %)	6 (10.5 %)	
Intravenous hydration type	None	2 (100 %)	0 (0.0 %)	0.231
	0.9% sodium chloride (NaCl)	3 (75.0 %)	1 (25.0 %	
	Isotonic saline administered 12 hours before the procedure	64 (92.8 %)	5 (7.2 %)	
	Others	23 (82.1 %)	5 (17.9 %)	
Hydration time	Pre-TAVI	64 (92.8 %)	5 (7.2 %)	0.121
	Post-TAVI	15 (88.2 %)	2 (11.8%)	
	Both	13 (76.5 %)	4 (23.5 %)	
Contrast media volume administered, mean± SD		192.62 ± 82.20	209.36 ± 87.75	0.527
Mehran score	Low risk (0 to 5)	21 (100.0%)	0 (0.0%)	0.120
	Moderate risk (6 to 10)	33 (91.7 %)	3 (8.3 %)	
	High risk (11 to 15)	26 (83. 9%)	5 (16.1 %)	
	Very high risk (≥16)	12 (80.0 %)	3 (20.0 %)	

Procedural data 

The analysis in Table 4 shows that the indication for TAVI in both the AKI (-) and AKI (+) groups did not result in significant differences (p = 1.00). Furthermore, the study determined that the type of anesthesia used had no significant impact on outcomes. The types of valves utilized during the TAVI procedures in our study included the Evolut-R™ (Medtronic, Minneapolis, MN), the ACURATE-Neo™ (Boston Scientific, Marlborough, MA), and the SAPIEN-3™ (Edwards Lifesciences, Irvine, CA). The Evolut-R™ and ACURATE-Neo™ are self-expanding valves, while the SAPIEN-3™ is a balloon-expandable valve. Notably, the type of valve used demonstrated a significant relationship with the occurrence of AKI (P= 0.005).

**Table 4:** Procedural data AKI: acute kidney injury; TAVI: transcatheter aortic valve implantation

Procedural Data		AKI (-); n (%)	AKI (+); n (%)	p-value
Indication of TAVI	Severe aortic valve stenosis	77 (88.5%)	10 (11.5 %)	1.00
	Severe symptomatic aortic valve stenosis	13 (92.9%)	1 (7.1 %)	
	Severe symptomatic aortic valve prosthesis degenerative	2 (100.0 %)	0 (0.0 %)	
Type of anesthesia	General anesthesia	83(88.3%)	11 (11.7%)	0.592
	Local anesthesia with sedation	9 (100.0%)	0 (0.0 %)	
Type of valve	Evolut-R™(Medtronic)	79 (94.0%)	5 (6.0%)	0.005
	SAPIEN-3™ (Edwards Lifesciences)	10 (71.4 %)	4 (28.6 %)	
	ACURATE-Neo™ (Boston Scientific)	3 (60.0 %)	2 (40.0 %)	

## Discussion

A combination of procedural and patient-related factors makes AKI a significant complication following TAVI. This underscores the criticality of monitoring and managing renal function in TAVI patients. Knowledge about the incidence of AKI allows healthcare providers to take preventive action against it and improve outcomes for these patients. In our study, the development of AKI within seven days after the procedure was seen in 11 (10.7%) patients. This finding is similar to that obtained by Julien et al. [26], who reported that out of 107,814 individuals from the United States who underwent TAVI, 11,566 (10.7%) experienced AKI. According to another study conducted in Germany, there were cases of AKI among 111 (13.8%) out of 804 patients who underwent TAVI [10]. On the other hand, a higher incidence conducted in a local study to determine the occurrence of AKI following TAVI found that 14 (17.3%) out of 80 patients with AKI were reported by Alatawi et al. [27]. Additionally, Alhamad et al. [28] elucidated that preexisting chronic kidney conditions significantly contribute to the incidence of AKI post TAVI. Echoing this, our research further identifies existing renal conditions, specifically CKD, as crucial determinants. We also found a significant association between hyperlipidemia and the development of AKI post TAVI. This may be due to the link between hyperlipidemia and degenerative aortic valve stenosis [29] or as suggested by Scherner et al. [7] it may be due to the formation of atherosclerotic emboli generated during valvuloplasty when the passage of the catheter through the aorta and deployment of the valve prosthesis may play a role in decreasing the eGFR following TAVI. Recognizing these risk factors is imperative for enhancing patient care protocols and robust postoperative monitoring frameworks, thereby mitigating the associated risks of TAVI. 

Secondly, some laboratory tests such as hemoglobin, white blood cell count, platelet count, and electrolyte levels showed no significant differences between patients with and without AKI, suggesting their limited role in AKI development post TAVI. Conversely, the study demonstrates that specific laboratory parameters, specifically sCr, eGFR, and BUN, significantly impacted patient outcomes on the procedure date and post TAVI. This is expected since these tests are reliable indicators of kidney function and can be used to assess the impact of contrast media on the kidneys. Similarly, Sudarsky et al. and Molen et al. identified eGFR as a significant predictor of AKI [5, 20]. Moreover, our research identified a significant association between the type of valve used and the occurrence of AKI. This finding correlates with the study by Loizzi et al. [30], which reported a higher incidence of AKI in patients undergoing TAVI with a specific type of valve, which is self-expanding prostheses. The increased AKI risk may be attributed to the extended periods of extreme hypotension required for the deployment of self-expanding valves compared to balloon-expandable valves. However, further clinical studies are necessary to confirm this association [30].

Thirdly, although there is an ongoing debate about the ideal hydration protocol, Molen et al.'s [20] study emphasized hydration with normal saline or sodium bicarbonate as the primary method of preventing PC-AKI. Nevertheless, our evaluation of preventative measures such as hydration did not significantly affect the occurrence of AKI. This discrepancy might be due to different protocol strategies used to hydrate the patients, which will prompt further research into optimizing preventive measures to reduce the risk of AKI post TAVI. Furthermore, neither the dose of contrast media administered nor the Mehran score significantly correlated with AKI. This lack of significant correlation could be attributed to the small sample size. Overall, the study's findings stress the need for personalized risk assessment and management strategies to reduce the risk of AKI following TAVI, particularly in patients with hyperlipidemia and preexisting kidney conditions.

Limitations

This study has some limitations. Firstly, the retrospective design limits the ability to establish causality between the analyzed factors and the development of AKI. Secondly, the sample size is relatively small, which may limit the generalizability of the findings. Additionally, the study relied on data collected from medical records, which may be subject to incomplete or inaccurate documentation. Finally, the study focused on a specific population undergoing TAVI, which may limit the generalizability of the findings to other patient populations.

## Conclusions

Acute kidney injury remains a common and concerning complication following TAVI. The study found that 11 (10.7%) patients who underwent TAVI experienced AKI seven days after the TAVI procedure. The study also highlights the significance of identifying patients with hyperlipidemia and pre-existing kidney conditions and closely monitoring renal function. Though some preventive methods did not significantly affect the occurrence of AKI, further research may be needed to refine the preventive strategies. 
